# 4D Graph-Based Segmentation for Reproducible and Sensitive Choroid Quantification From Longitudinal OCT Scans

**DOI:** 10.1167/iovs.15-18924

**Published:** 2016-08

**Authors:** Ipek Oguz, Michael D. Abramoff, Li Zhang, Kyungmoo Lee, Ellen Ziyi Zhang, Milan Sonka

**Affiliations:** 1 Department of Ophthalmology and Visual Sciences, The University of Iowa, Iowa City, Iowa, United States; 2 Iowa Institute for Biomedical Imaging, The University of Iowa, Iowa City, Iowa, United States; 3 Department of Radiology, University of Pennsylvania, Philadelphia, Pennsylvania, United States; 4 Veterans Affairs Medical Center, Iowa City, Iowa, United States; 5 Wellman Center for Photomedicine, Massachusetts General Hospital and Harvard Medical School, Boston, Massachusetts, United States

**Keywords:** image segmentation, choroid, OCT, multilayered graph search

## Abstract

**Purpose:**

Longitudinal imaging is becoming more commonplace for studies of disease progression, response to treatment, and healthy maturation. Accurate and reproducible quantification methods are desirable to fully mine the wealth of data in such datasets. However, most current retinal OCT segmentation methods are cross-sectional and fail to leverage the inherent context present in longitudinal sequences of images.

**Methods:**

We propose a novel graph-based method for segmentation of multiple three-dimensional (3D) scans over time (termed 3D + time or 4D). The usefulness of this approach in retinal imaging is illustrated in the segmentation of the choroidal surfaces from longitudinal optical coherence tomography (OCT) scans. A total of 3219 synthetic (3070) and patient (149) OCT images were segmented for validation of our approach.

**Results:**

The results show that the proposed 4D segmentation method is significantly more reproducible (*P* < 0.001) than the 3D approach and is significantly more sensitive to temporal changes (*P* < 0.0001) achieved by the substantial increase of measurement robustness.

**Conclusions:**

This is the first automated 4D method for jointly quantifying choroidal thickness in longitudinal OCT studies. Our method is robust to image noise and produces more reproducible choroidal thickness measurements than a sequence of independent 3D segmentations, without sacrificing sensitivity to temporal changes.

Longitudinal studies of the retina and choroid using series of optical coherence tomography (OCT) images offer a uniquely powerful view into the dynamic changes of retinal and choroid anatomy over the course of a study.^1–4^ Compared with the large intersubject variability in cross-sectional studies, within-subject measurements allow more precise quantification of change over time. This is of great interest both for quantifying disease progression and in studies of aging.

Currently existing image analysis methods for OCT do not fully leverage the available data in such studies. Most existing approaches are either two-dimensional (2D) or 3D^4–8^ and treat the OCT images from different time points completely independently. These methods rely on statistical analysis for exploring the relationship of the time points with each other. Recently, a new approach that considers the longitudinal time sequence during image analysis was proposed^9^; however, this method appears to suffer from temporal over-regularization as it can only detect small temporal changes. Such over-regularization is a concern in many longitudinal analysis methods, which aim to reduce the measurement discrepancies between time points by using temporal regularization; such over-regularization can be seen as preferring no or limited changes over time for which the allowed changes would not reflect the true changes of anatomy/pathology over time. Although temporal regularization is indeed useful to reduce measurement noise and increase reproducibility, in the extreme cases, over-regularization would result in an inability to detect actual temporal changes present in the data, which is clearly undesirable. In summary, no method currently exists for a successful 4D (3D + time) image quantification of longitudinal OCT scans that adequately balances the need to detect small changes over time with the need for consistent measurements between time points.

We present a novel 4D approach for simultaneous graph-based segmentation in longitudinal studies. It is capable of simultaneously segmenting multiple surfaces from all the time points associated with a subject in a globally optimal manner while satisfying the topology and smoothness constraints specified at each time point.

We illustrate this new method in application to the segmentation of the choroid. The choroid is the vascular plexus between the retina and the sclera that forms a vascular system, which is crucial for oxygenation and metabolic activity of the retina.^10,11^ Choroidal thickness is an important retinal biomarker that is affected in many diseases of the retina, for example, in AMD,^12^ in response to anti-VEGF therapy in diabetic macular edema,^13^ and in healthy aging.^14^ However, because of absorption by the RPE, the choroid is prone to low signal-to-noise ratio (SNR) in OCT scans and is therefore challenging to segment,^15–18^ even in swept-source OCT, which is designed to increase SNR in the deeper layers. This is especially problematic in longitudinal studies where small changes in the noise appearance between images from different time points may lead to highly inconsistent surface positioning between time points, reducing statistical power in studies.

Validation of the proposed algorithm is done using patient OCT scans and synthetic experiments. We show that the 4D approach successfully leverages the inherent redundancy in longitudinal image series to reduce measurement noise and leads to better automated quantification of OCT images. As discussed above, an important concern for longitudinal segmentation algorithms is the danger of over-regularization in the temporal domain; we demonstrate that our approach successfully enforces temporal coherence without overly regularizing by showing its effectiveness in detecting temporal differences.

## Methods

### Subjects and Data Collection

Longitudinal sequences of spectral-domain (SD)-OCT images from a cohort of 15 AMD patients (age: 78.2 ± 9.6 years) diagnosed with choroidal neovascularization were used for the sensitivity experiment and the synthetic experiments. Age-related macular degeneration and its complications are not the subject of this study on 4D choroidal segmentation; rather, this specific cohort was chosen because of the large number of well-characterized OCT images that are available.

The image acquisition was done while the patients underwent an initial 12-week induction phase of anti-VEGF treatment, during which injections and imaging were uniform for all patients. The induction phase was defined as the process of administration of three intravitreal injections at 4-week intervals (weeks 0, 4, and 8) supplemented by OCT imaging sessions every 2 weeks for a total of seven scans per patient, or 105 OCT volumes overall.

Image acquisition was performed using a Topcon swept-source OCT scanner (Topcon, Paramus, NJ, USA), centered on the fovea of the retina. The device acquires anisotropic 3D OCT images having 512 × 128 × 885 voxels with 11.72 × 46.88 × 2.6-μm^3^ spacing and covering a volume of 6 × 6 × 2.3 mm^3^.

A second set of images was obtained from a separate cohort of 22 glaucoma patients that were scanned twice within a 3-month period for a reproducibility study (average time between two scans: 44 ± 27 days), for a total of 44 3D OCT volumes. This dataset was used in the reproducibility experiment. Image acquisition was performed using a Heidelberg Spectralis OCT scanner (Heidelberg Spectralis; Heidelberg Engineering, Heidelberg, Germany), centered on the fovea of the retina. The device acquires anisotropic 3D OCT images of 61 × 768 × 496 voxels with 127.193 × 11.291 × 3.872-μm^3^ spacing, and covering the volume of 7.76 × 8.67 × 1.92 mm^3^.

This research followed the tenets of the Declaration of Helsinki. Written informed consent was obtained from the subjects after explanation of the nature and possible consequences of the study. The research was approved by the institutional human experimentation committee or institutional review board.

### Initialization

The location of Bruch's membrane (BM) is needed as input for initializing the choroidal segmentation approach. For this purpose, the OCT volumetric images were segmented individually using our previously reported Iowa Reference Algorithm that yielded 11 intraretinal surfaces.^6,19–23^ Because the retinal angle between the incident light and subject's optical axis varied in OCT volumetric images, some geometric distortion occurs in the choroidal layer. An angle adjustment approach^24^ was applied to the original OCT volumes to overcome this. Bruch's membrane was transformed to a relatively symmetrical surface in the OCT images, and then the BM was modeled as a convex arc on each B-scan, and the curvature of this arc was computed using the average axial length of a human eyeball. A single surface graph-search method was used to segment BM by utilizing this arc model as shape-prior information.^19^ The OCT volumes were then flattened using the BM surface. The flattened scans from all time points are aligned such that the horizontal line representing the (flat) BM surface is at the same position in each volume. No within-plane alignment was necessary in the presented experiments because all scans were carefully centered on the fovea; however, other datasets with greater foveal location variability may need this as an additional preprocessing step.

The resulting flattened images and the BM surfaces were used as input to both the cross-sectional (3D) and longitudinal (4D) segmentation approaches described below.

### Prior Work: 3D Graph Segmentation

The 3D segmentation of the choroid^4,8^ was done using the LOGISMOS framework.^25^ We begin by cropping the flattened OCT image to a sufficiently large region of interest underneath the BM surface. The empirically determined size of the crop region was 489 μm (188 voxels) in the anterior–posterior direction. Twenty-one micrometers of this region was anterior to the BM surface, to allow for recovery from any errors in the BM initialization. For computational efficiency, as well as to alleviate the anisotropy of the acquisition protocol, this cropped image was down-sampled by a factor of (2 × 4 × 1) in the inferior–superior, anterior–posterior, and nasal–temporal directions, respectively. A median filter was used in a one-voxel neighborhood to reduce noise.

A multisurface graph-cut approach was used to jointly segment the lower and the upper boundary of the choroid region. For this purpose, we constructed a graph such that each image voxel is represented by a graph node. Neighboring voxels in the anterior–posterior direction are grouped together in graph columns, connected to each other via intracolumn arcs. Neighboring voxels in the nasal–temporal and inferior–superior directions are represented by neighboring graph columns, connected to each other by intercolumn arcs, which encode hard and soft constraints for spatial smoothness. Two identical copies of this graph were created to represent the two surfaces of the choroid; these two graphs are connected to each other via intersurface arcs, which encode the minimum and maximum allowed distance between the two surfaces. An edge-sensitive image derivative filter along the posterior–anterior direction was used to determine the cost at each graph voxel. The graph thus constructed was optimized using the LOGISMOS framework^25^ to obtain the optimal surface segmentations.

### Proposed Method: 4D Graph Segmentation

The proposed 4D method jointly segments the upper and lower surfaces of the choroid from *N* time points, that is, 2*N* surfaces are segmented simultaneously. The preprocessing of each image, including the flattening, cropping, and down-sampling, was identical to the 3D approach.

Because the graph construction for the 4D segmentation assumes spatial correspondence between the images from different time points, the images were first registered to each other. The anterior–posterior registration was provided implicitly by the image flattening step, based on the BM surface. For in-plane registration, all images were centered on the patient's fixation.

The graph construction for the 4D segmentation was different than the 3D approach. Each image was represented by a subgraph constructed as described in the 3D segmentation, using the intracolumn, intercolumn, and intersurface arcs. These subgraphs were connected to each other using novel inter–time point arcs to encode temporal constraints between consecutive time points. In particular, for each vertex 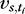 (*x*,*y*,*z*), where *s* denotes the surface ID (lower or upper choroid), *t_i_* denotes the *i*th time point, and (*x*, *y*, *z*) denotes the spatial coordinates, the following infinite-weighted arcs were added:

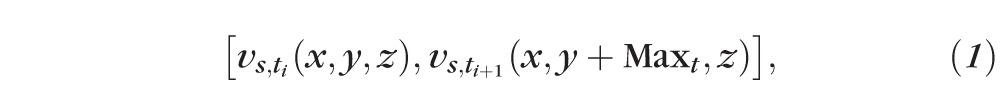


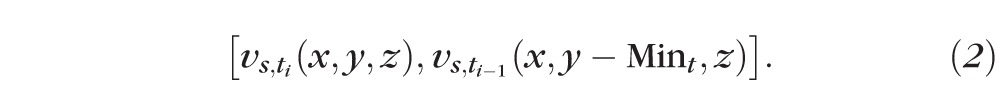



Equation 1 encodes the maximum allowed vertical displacement constraint between two consecutive time points, whereas Equation 2 encodes the minimal allowed vertical displacement constraint. For the choroid, for all presented experiments, Min*_t_* and Max*_t_* were set to [−2,2] node intervals for the upper choroid surface and [−5,5] node intervals for the lower choroid surface. These empirically determined settings indicate that each surface can move anteriorly or posteriorly independently. The choroid region bound by these two surfaces can thus thin or thicken by up to seven node intervals. Because the graph is constructed in the down-sampled image space, this corresponds to 72.8-μm thinning or thickening between consecutive time points, which is clinically reasonable.

It is important to realize that the arcs used to enforce these temporal constraints carry infinite weight and are therefore hard constraints, that is, thresholds on what is considered a feasible solution of the graph. This is in contrast to soft constraints, represented by finite-weighted arcs, which penalize large displacements. Such soft temporal constraints were avoided in our approach as they introduce a segmentation bias by preferring smaller displacement, which may reduce the temporal sensitivity of the segmentation algorithm.

We note that the temporal constraint for the upper surface was chosen to be more strict because the spatial registration was based on the BM surface, which is immediately next to this upper choroid surface. The lower choroid surface, which is farther away, is assigned a more relaxed temporal regularization threshold.

Given *N* longitudinal OCT volumes from *N* time points, all *N* subgraphs are connected to each other using the inter–time point arcs described above, to result in a single complex graph that represents the segmentation problem for all *N* time points. The segmentation is then carried out using a single graph-cut on this complex graph to achieve simultaneous segmentation of the 2*N* surfaces.

### Reproducibility Experiment on Synthetic Data

To assess the reproducibility and robustness to noise of our novel 4D choroid segmentation method, we conducted a simulation study. Starting with patient OCT images, increasing levels of random speckle noise was synthetically introduced. The choroid was segmented independently (3D) and jointly with the proposed 4D method from pairs of noisy images. We report both the surface positioning reproducibility and choroid thickness reproducibility.

#### Speckle Noise Simulation.

The primary type of noise in OCT images, which derives from the low coherence light source, is speckle,^26^ which is equivalent to additive Gaussian noise in the linear scale. To simulate noisy OCT images, we first converted the real 16-bit OCT images to decibel scale, by linearly scaling the intensities such that the voxel intensity range corresponded to the dynamic range of the OCT camera. The dynamic range of our OCT camera was empirically determined to be 42 dB. Then, the image was de-logged to convert it to linear scale, according to

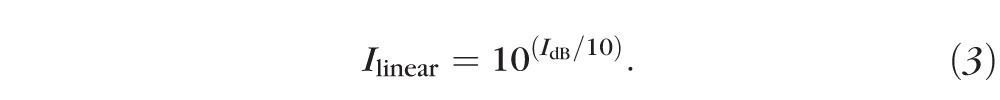



In this linear scale, additive Gaussian noise of the desired SD was introduced to the image. Finally, the images were brought back to their original scale, by first re-logging them according to

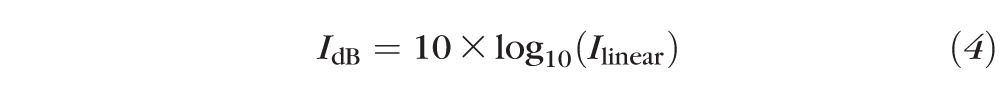
and then scaling them back up to 16-bit OCT image range.

#### Dataset.

The OCT scans from the first time point for each of the 15 AMD patients were chosen as the starting points for this experiment. For each subject, for each noise level, 10 noisy copies of the image were created by introducing randomized speckle noise. These 10 images were split into five pairs. Each such pair was segmented both independently in 3D and jointly in 4D. This process was repeated for 13 different noise levels (σ = 1, 3,…, 25), where the noise level is the SD of the Gaussian additive noise in the linear scale as described above. A total of 15 × 0 × 13 = 1950 synthetic images were thus used in this experiment. For a fair comparison, the initial BM surface was computed only once per subject, on the real image (before addition of any speckle noise); this surface was used as input to both 3D and 4D segmentation methods.

#### Analysis.

Because the underlying retinal and choroidal anatomy is the same for a given pair, these pairs can be considered as a test–retest setup; therefore, the segmentation results should be ideally identical. Thus, any discrepancies are purely due to noise and are reported as reproducibility error. In particular, we report the reproducibility error associated with the surface positioning of the lower choroid boundary and choroid thickness. Paired *t*-tests between 3D and 4D segmentation reproducibility errors are used to assess statistical significance. We also present scatter plots of test–retest thickness measurements and report the correlation between these values at each noise level for 3D and 4D.

Complete segmentation failures were excluded from analysis. We empirically determined that an average reproducibility error of >100 μm indicated such cases.

### Temporal Sensitivity Experiment on Synthetic Data

To assess the sensitivity of our novel 4D choroid segmentation method to detect temporal changes, we conducted a synthetic simulation study. Starting with patient OCT images, random amounts of deformation in the anterior–posterior direction were added to the choroid and surrounding regions to simulate thinning and thickening, which may happen in real data from reasons such as disease progression, drug effects, or aging. The choroid was segmented independently (3D) and jointly (4D) from the resulting images. Because the artificially introduced deformation amount is known, we use this dataset to quantify the sensitivity of each algorithm.

#### B-Spline Deformation.

**Figure 1:**
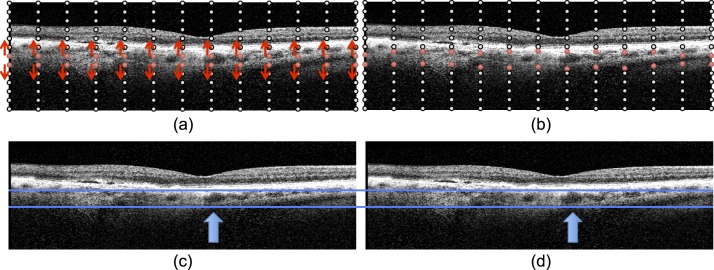
B-spline deformation method for the synthetic sensitivity experiments. (**a**) A regular grid 13 × 13 × 13 B-spline node is created. The nodes that approximately correspond to the choroid region (*red circles*) are deformed, whereas the other nodes remain stationary (*black circles*). (**b**) For thickening simulations (positive *α*), the B-spline nodes in the choroid region move away from each other vertically (shown), whereas for thinning simulations (negative *α*), they move toward each other. Note that the local deformation amount is randomly selected, allowing more realistic local thinning/thickening effects rather than a global shrinking of the choroid at a constant rate. (**c**, **d**) Thickening simulation example. (**c**) B-scan from the original image. (**d**) The same B-scan with a synthetically thickened choroid. *Blue arrows* highlight a visibly enlarged vessel, whereas other regions of the B-scan show more modest amounts of deformation.

Given a real OCT image, a series of eight synthetically deformed images was created for each trial, representing a sequence of eight time points. At each time point *t_i_* (*i* = 0…8, with *t*_0_ designating the real OCT image), a target thinning rate *α_i_* was generated as a random sample from a uniform distribution between [−25,25] μm, with a negative *α* denoting a thinning choroid and a positive *α* denoting a thickening choroid. These bounds for *α_i_* were chosen to represent a wide range of changes that can be realistically observed in the choroid. A B-spline grid with 13 nodes in each dimension is created to represent the whole OCT image domain. This grid density was empirically chosen to give adequate resolution, such that the choroid and surrounding regions can be manipulated separately from the rest of the image domain. The B-spline deformation was set to 0 outside the choroid region of interest (ROI), which is represented by two rows of grid nodes around the choroid. The B-spline grid is illustrated in Figure 1a. Inside the choroid region, each grid node (*x*,*y*) was assigned a local thinning amount *β_i_*_,(_*_x_*_,_*_y_*_)_, which was randomly drawn from a uniform distribution between [0,2|*α_i_*|]. Then, the vertical displacement at the grid node corresponding to the top row of the choroid ROI was set to *β_i_*_,(_*_x_*_,_*_y_*_)_, and at the grid node corresponding to the bottom row of the choroid ROI, it was set to −*β_i_*_,(_*_x_*_,_*_y_*_)_, for positive *α_i_*. The vertical displacement values were reversed for negative *α_i_*. We note that this corresponds to a local displacement of 2*β_i_*_,(_*_x_*_,_*_y_*_)_. Figure 1b shows these local displacements of the B-spline nodes. The smooth deformation field represented by the B-spline simulation ensures that the region surrounding the true choroid is stretched or compressed to accommodate the desired motion of the choroid itself.

At each time point *t_i_*, the random deformation field thus computed was applied to the image at *t_i_*_−1_. Figures 1c and 1d show the thickening simulation for a particular B-scan from the experiment. We note that the *α_i_* are independent from each other; in practice, this means a time sequence can have alternating thinning and thickening periods, at varying rates of deformation. We chose this approach rather than consistent thinning or consistent thickening to obtain a wider range of situations that may occur in real clinical data based on disease progression and treatment schedule.

#### Dataset.

Similar to the reproducibility experiment, the real OCT scans from the first time point for 14 AMD patients were used as the starting points *t*_0_ for the sensitivity experiment. One subject was excluded from this study as it had a very thin choroid, which occasionally led the choroidal layer to collapse to a line locally when a random deformation was applied. This resulted in an inability to create a synthetic 4D dataset and does not indicate a choroidal thickness measurement limitation of the reported image segmentation method. For each subject, 10 trials were conducted. For each trial, eight randomly deformed images were generated as described above. These images at *t*_1,…,8_ were segmented both independently in 3D and jointly in 4D. A total of 14 × 10 × 8 = 1120 synthetic images were thus used in this experiment. For a fair comparison, the initial BM surface was computed only once for each deformed image and used as input to both 3D and 4D segmentation algorithms.

#### Analysis.

The known synthetic deformation field is used to compute the ground truth for choroid thickness change *δ*_true_(*i*) between time points *t_i_* and *t_i_*_−1_, by summing the local *β_i_*_,(_*_x_*_,_*_y_*_)_s. It is important to note that, although these relative thickness changes are known between time points, the absolute choroid thickness at any given time point is unknown, because we do not have the ground truth at *t*_0_. For assessing the temporal sensitivity of the two methods, we compute the choroid thickness at each time point using the 3D and 4D segmentation results, and we report *δ*_3D_(*i*) − *δ*_true_(*i*) and *δ*_4D_(*i*) − *δ*_true_(*i*) as momentary sensitivity errors. Both signed and unsigned differences are reported. In this context, signed error represents a measurement bias in sensitivity, whereas unsigned error represents the bulk measurement error. In addition to these momentary thickness changes, we also consider cumulative thickness changes, Δ_true_(*i*), Δ_3D_(*i*), and Δ_4D_(*i*), which represents the total change between time points *t_i_* and *t*_0_, computed as 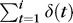 . Paired *t*-tests are used to statistically compare the findings.

### Glaucoma Cohort for Reproducibility

To assess the reproducibility achieved by our algorithm in a clinical dataset, we leveraged the test–retest glaucoma dataset. For each of the 22 subjects in this cohort, the two OCT volumes were segmented cross-sectionally (3D) and longitudinally (4D). The comparison of the results from the two approaches offers additional insights regarding the effect of the temporal constraints proposed in our algorithm. Given the short time period between the two scan acquisitions and the very slowly progressing character of the glaucoma disease, no changes to the underlying choroidal anatomy are expected within 3 months for any given subject. Thus, similar to the synthetic reproducibility experiment we presented earlier, any thickness discrepancies would be purely due to noise and are reported as thickness reproducibility error. Similar to the synthetic experiment, we present scatter plots of test–retest thickness measurements and report the correlation between these values and the respective regression equations.

### Age-Related Macular Degeneration Cohort for Sensitivity

Finally, to assess the performance of our algorithm in a clinical dataset with respect to sensitivity, we analyzed the longitudinal AMD data. For each of the 15 subjects in the study, the 7 OCT images were segmented cross-sectionally (3D) and longitudinally (4D), for a total of 105 images. The resulting choroidal thickness measurements are compared between the two methods, including the mean choroidal thickness (over time points) per patient, the SD of choroidal thickness (over time points) per patient, and the slope of the temporal change (i.e., change in thickness divided by total study time) per patient. This temporal slope represents an estimate of the true anatomical change, whereas the SD of choroid thickness measurement between time points (for a given subject) represents a combination of measurement noise and true anatomical change. Our hypothesis was that longitudinal 4D segmentation would result in lower noise (smaller SD) but will retain its temporal sensitivity (same slope). Paired *t*-tests were used for statistical comparison.

## Results

### Reproducibility Experiment on Synthetic Data

**Figure 2:**
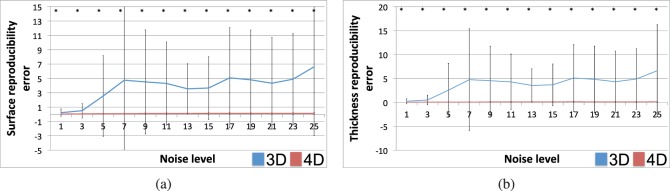
Choroid segmentation reproducibility in the presence of simulated speckle noise. (**a**) Lower-choroid surface position reproducibility error (μm) per noise level (*σ* of Gaussian kernel). Longitudinal (4D, *red*) segmentation resulted in significantly more reproducible surfaces (*P* < 0.001) than cross-sectional (3D, *blue*) segmentation for each tested noise level. (**b**) Choroid thickness reproducibility error (μm) per noise level. Four-dimensional segmentation led to significantly more reproducible thickness measurements (*P* < 0.001) than 3D segmentation for each tested noise level. Note that the *error bars* for the 4D plots are difficult to see because of their small scale.

Figure 2 presents the results of the reproducibility experiment. Given the pairs of synthetically generated noisy images, Figure 2a presents the reproducibility error in the lower choroid boundary location, whereas Figure 2b presents the reproducibility error in choroid thickness measurement. The asterisk denotes highly significant statistical differences (*P* < 0.001) between 3D and 4D segmentation methods; 4D segmentation was significantly more reproducible than 3D segmentation at each tested noise level, in terms of both surface positioning and thickness measurement. For eight pairs of images, 3D segmentation results were excluded due to segmentation failure.

**Figure 3:**
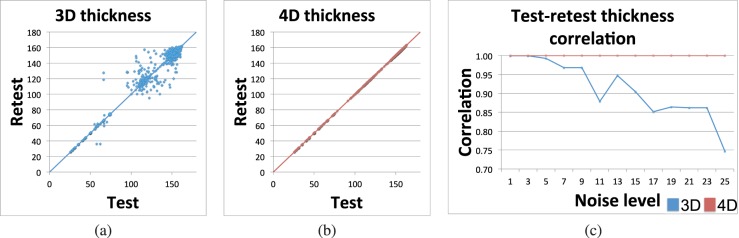
Choroid thickness consistency in the presence of simulated speckle noise. (**a**) Test–retest choroid thickness measurements (μm) for cross-sectional (3D) segmentation. *y* = *x* line is shown for reference. (**b**) Test–retest choroid thickness measurements (μm) for longitudinal (4D) segmentation. *y* = *x* line is shown for reference. (**c**) Correlation of test–retest choroid thickness measurements per noise level (σ of Gaussian kernel). High correlation corresponds to highly consistent measurements. Four-dimensional (*red*) measurements were significantly more consistent than 3D (*blue*) measurements (*P* < 0.002).

Figure 3 presents the consistency of the thickness measurements in the reproducibility experiment. For each pair of noisy images, the average choroid thickness for the first image is plotted against the average choroid thickness for the second image. Figure 3a presents this test–retest scatter plot for cross-sectional (3D) segmentation, whereas Figure 3b shows longitudinal (4D) segmentation results; results from all noise levels are pooled together for this figure. The dramatic improvement in measurement consistency afforded by 4D segmentation is evident from these plots. Figure 3c presents the correlation between these test–retest thickness measurements per noise level. The correlation measures for 4D segmentation were significantly higher (*P* < 0.002) than for 3D segmentation.

### Temporal Sensitivity Experiment on Synthetic Data

**Figure 4:**
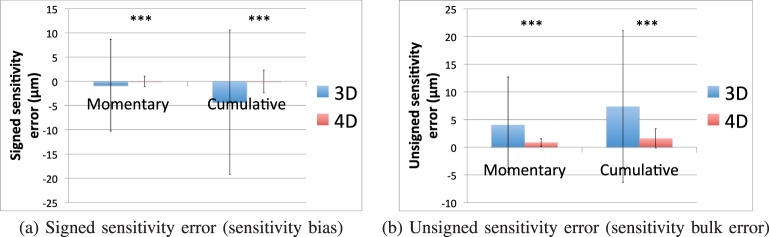
Sensitivity error. In the temporal sensitivity experiment where the choroid is synthetically deformed, we report the detected temporal thickness change compared to the ground truth (the true amount of vertical deformation synthetically introduced) as the sensitivity error. Four-dimensional segmentation was highly significantly more sensitive than 3D segmentation in all compared measurements (*P* < 0.001 in paired *t*-tests).

The Table and Figure 4 summarize the results of the sensitivity experiment using the synthetic deformation fields. For both signed and unsigned error metrics, the 4D approach was significantly more sensitive to temporal change than the 3D approach (*P* < 0.001), both for momentary and cumulative time intervals.

**Table:**
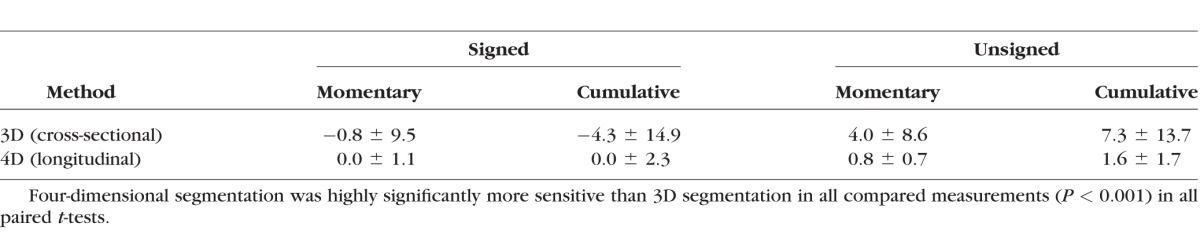
Sensitivity Error

**Figure 5:**
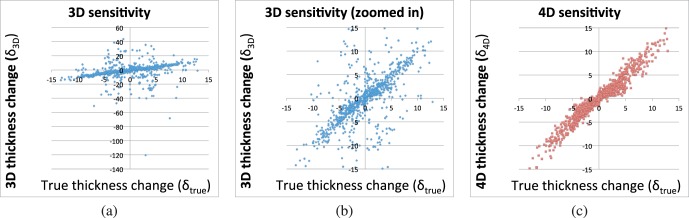
Sensitivity to temporal changes in choroidal thickness. All units are micrometers. (**a**) Changes in choroid thickness measurements for cross-sectional (3D) segmentation compared with the known synthetic deformation amount. The same data is presented in **b** after thresholding and zooming to the [−15,15]-μm range, for a fair comparison with 4D. (**c**) Changes in choroid thickness measurements for longitudinal (4D) segmentation compared with the known synthetic deformation amount. The Pearson correlation coefficients were *R*^2^ = 0.299 for 3D and *R*^2^ = 0.970 for 4D.

Figure 5 shows the scatter plots of the automatically measured momentary choroid thickness changes against the known deformation amounts, using the 3D and 4D approaches. The correlation between the momentary *δ*_3D_ and *δ*_true_(*i*) was *R*^2^ = 0.299, compared with a correlation of *R*^2^ = 0.970 between momentary *δ*_4D_ and *δ*_true_(*i*). Similarly, the correlation between the cumulative Δ_3D_ and Δ_true_(*i*) was *R*^2^ = 0.288 compared with a correlation of *R*^2^ = 0.975 between cumulative Δ_4D_ and Δ_true_(*i*).

**Figure 6:**
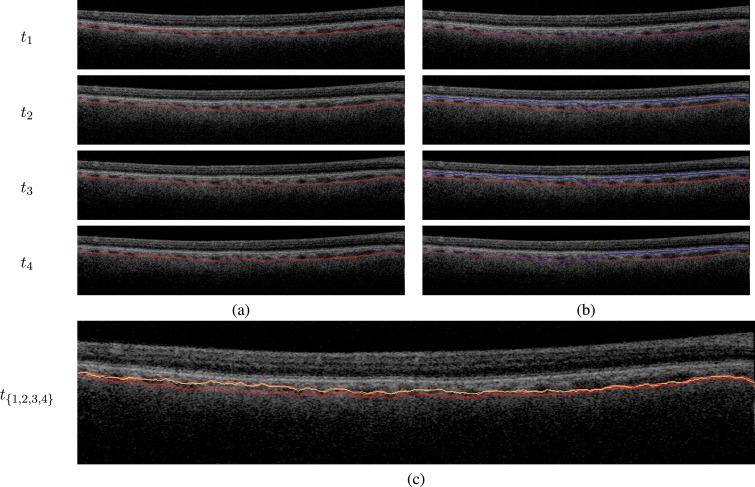
Choroid segmentation in a synthetically deforming time sequence. Four consecutive time points are shown in subsequent rows. (**a**) Deformed OCT image at that time point. The choroid surface at *t*_4_ is overlaid on each image to help highlight the temporal changes. The *t*_1_ image can be appreciated to have a thicker choroid than the *t*_2_−*t*_4_ images, especially on the nasal side. (**b**) The 3D (*blue*) and 4D (*red*) lower choroid surfaces at each time point, overlaid with the deformed OCT image at that time point. The two methods closely agree in the *t*_1_ image. At *t*_2_ and *t*_3_, the 3D segmentation fails throughout most of the B-scan (except in the center) and captures the upper boundary of the choroid rather than the lower boundary. At *t*_4_, the 3D segmentation is successful in only the temporal side of the image and locks onto the wrong surface in the nasal side. Four-dimensional segmentation (*red*) leverages temporal regularization to successfully segment all four time points. (**c**) The 4D segmentations from each time point are overlaid on the *t*_1_ OCT image (*different shades of red* represent different time points). It can be clearly seen that the *t*_1_ segmentation is substantially different than the *t*_2_−*t*_4_ segmentations, with more pronounced thickening in the temporal side than in the nasal side, in accordance with the data. This illustrates that the temporal regularization is not an obstacle to sensitivity to temporal change actually present in the data.

Figure 6 presents the results qualitatively for a single trial. The first four randomly deformed images are shown in column a. The same surface is overlaid in red on these images to help assess the temporal change; it can be clearly seen that the image at *t*_1_ has a thicker choroid than the images at *t*_2_–*t*_4_, especially on the nasal side of the image. Quantitatively, the true change in thickness was *δ*_true_(1) = 12 μm, *δ*_true_(2) = 1 μm, and *δ*_true_(3) = −2 μm. The 3D and 4D segmentations for the lower choroid boundary are shown in column b. Although the two methods closely agree at *t*_1_, the 3D segmentation results are very poor at *t*_2_ and *t*_3_, where they capture the wrong surface, presumably due to local noise and poorly defined boundaries. At *t*_4_, the 3D segmentation captures the lower choroid surface on the nasal side of the image but again fails on the temporal side. The 4D segmentation leverages temporal regularization to generate reliably correct results at all four time points.

However, the temporal regularization does not prevent the 4D algorithm from capturing true temporal change present in the data. This is illustrated in Figure 6c, which shows the 4D segmentation results for the four time points overlaid on top of each other. It can be clearly seen that the lower choroid surface at *t*_1_ is substantially different than the later time points [*δ*_4D_(1) = 10 μm], in agreement with the *δ*_true_(1) value. In contrast, the thre surfaces corresponding to the time points *t*_2_–*t*_4_ are nearly identical, which agrees with the near-zero *δ*_true_ values for these time points. As shown in Figure 4, these anecdotal findings were also found to be statistically significant in quantitative analysis.

### Glaucoma Cohort for Reproducibility

**Figure 7:**
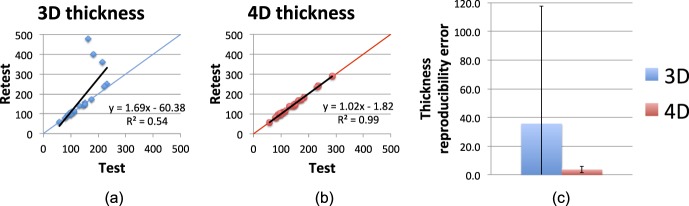
Choroidal thickness reproducibility in the glaucoma cohort. All units are micrometer. (**a**) Test–retest choroid thickness measurements (μm) for cross-sectional (3D) segmentation. The regression line is shown in *black*, and the *y* = *x* line is shown in *color* for reference. (**b**) Test–retest choroid thickness measurements (μm) for longitudinal (4D) segmentation. The regression line is shown in *black*, and the *y* = *x* line is shown in *color* for reference. (**c**) Thickness reproducibility error for the two methods. Mean and SD of absolute reproducibility error are shown for each method.

Figure 7 illustrates the results of the thickness reproducibility experiment in the glaucoma cohort. Figures 7a and 7b present the test–retest thickness measurements in the cross-sectional (3D) and longitudinal (4D) measurements, respectively. The test–retest correlation was *R*^2^ = 0.54 for the 3D approach (*y* = 1.69*x* − 60.38) and *R*^2^ = 0.99 for the 4D approach (*y* = 1.02*x* − 1.82). Note that despite allowing quite dramatic changes of surface positioning along the temporal direction (liberal temporal context constraints Min*_t_* and Max*_t_*), the slope and intercept of the regression line are virtually indistinguishable from 1 and 0, respectively. This further confirms both the hypothesized excellent performance of the 4D approach and also the validity of our expectation that choroidal thickness is not changing within 3 months in patients with glaucoma. Figure 7c further shows the thickness reproducibility error (i.e., the absolute difference between the test and retest thickness measurements). The mean and SD of the thickness reproducibility error was 35.6 ± 82.0 μm for the 3D approach compared with 3.7 ± 2.3 μm for the 4D approach. Similar to the results from the synthetic experiments, the dramatic improvement in reproducibility afforded by the longitudinal approach is clearly evident in these results.

### Age-Related Macular Degeneration Cohort for Sensitivity

In the AMD dataset, the mean choroidal thickness (50.6 ± 19.3 vs. 49.2 ± 15.1 μm) and temporal thinning rate (4.28 ± 3.11 vs. 7.43 ± 9.04 μm/12 wk) were not significantly different between the 3D and 4D methods (*P* > 0.34 for mean thickness, *P* > 0.22 for temporal slope). The SD of choroidal thickness was significantly different (*P* < 0.001, 2.43 ± 0.92 μm in 4D vs. 7.05 ± 3.69 μm in 3D). The results confirm our hypothesis that effective longitudinal (4D) analysis reduces measurement noise without sacrificing sensitivity to temporal change.

**Figure 8:**
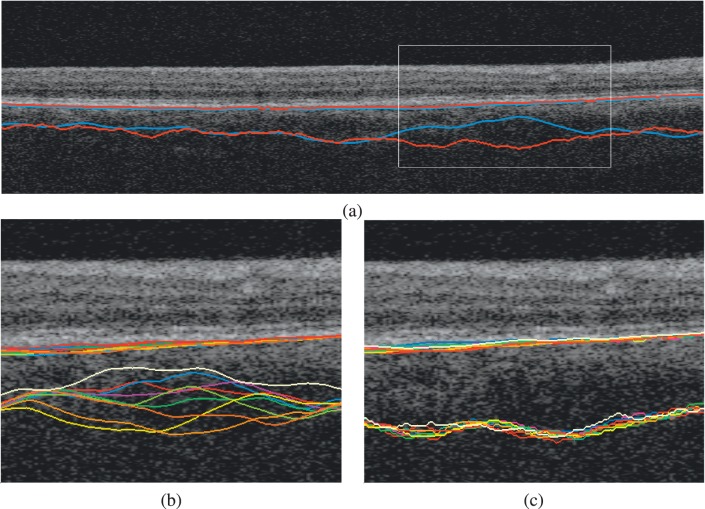
Choroidal segmentation consistency and accuracy. (**a**) Four-dimensional (*red*) and 3D (*blue*) segmentations are substantially different from each other in areas of high ambiguity (*white rectangle*). Note that the 3D approach erroneously cuts through choroidal vessels. **b** and **c** are zoomed in the region outlined by the *white rectangle* for visibility. (**b**) The 3D segmentations at the seven time points for the same subject. (**c**) The 4D segmentations at the seven time points for the same subject.

Figure 8 shows the 3D and 4D segmentation results qualitatively for a single subject. Although the 3D and 4D segmentations agree with each other in many places, image regions that present low signal or high noise lead to substantial discrepancies between the methods. In such regions, the 3D segmentation results may differ by up to 280 μm locally. Given the lack of evidence of true anatomical change to the choroid in the images, these changes are attributed to measurement noise of the 3D approach. The 4D joint segmentation resolves the discrepancies of 3D segmentation and offers segmentation consistency.

**Figure 9:**
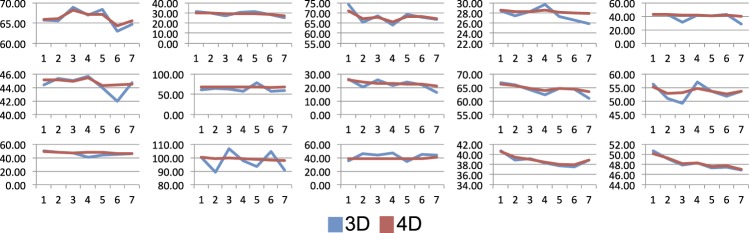
Choroidal thickness plots per subject. For each of the 15 patients in the AMD study, the average choroid thickness (*y* axis, μm) at each time point (*x* axis) is shown using the 3D and 4D quantification methods. Although the overall patterns are similar between the two methods, 4D measurements are visibly less noisy than their 3D counterparts due to the effective temporal regularization.

**Figure 10:**
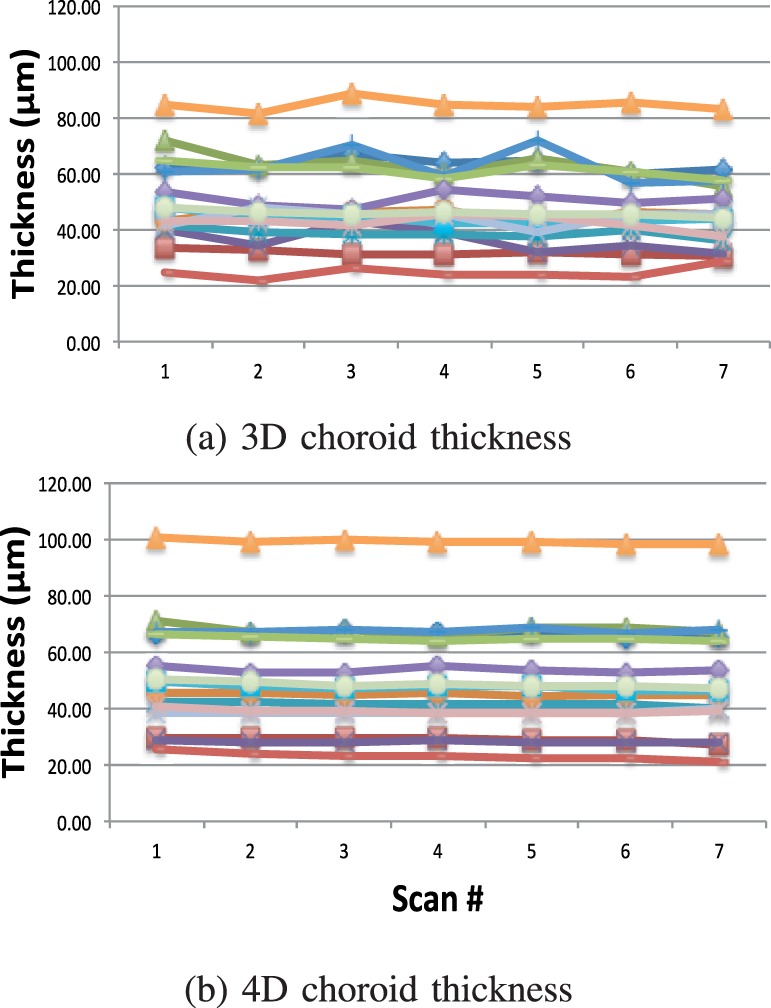
Average choroidal thickness computed in (**a**) 3D and (**b**) 4D. Colors denote individual patients. Both methods capture the choroidal thinning over the course of the study. Four-dimensional results show significantly smaller measurement variability over time.

Figure 9 shows the 3D and 4D thickness measurements per patient, over the course of the seven time points. The overall patterns are similar between the two methods, for example, the average choroid thickness of each patient and the amount of choroidal thinning over the study duration. However, the temporal 4D thickness functions are appreciably smoother than their 3D counterparts. These findings are also visible in Figure 10, where the same data are presented per method rather than per patient; different colors represent individual patients. Overall thinning is noticeable for both methods; however, 3D measurements are substantially noisier than their 4D counterparts.

The run time of the algorithm was roughly linearly related to the number of time points, and the additional overhead between 4D and 3D segmentation was relatively small. In particular, the run time for the subjects in the synthetic sensitivity study was 13 minutes 36 seconds ± 1 minute 14 seconds for eight scans in 3D (i.e., 1 minute 42 seconds ± 9 seconds for a single 3D scan) and 16 minutes 37 seconds ± 1 minute 43 seconds for 4D for the joint segmentation of all eight scans, representing less than 20% in computational overhead.

## Discussion

The presented results show that our novel 4D longitudinal segmentation algorithm is superior to its 3D counterparts. The temporal regularization afforded by this new method significantly increases the reproducibility of the results without sacrificing the sensitivity to detect temporal change; in fact, we found that the reduction in error leads to significantly higher temporal sensitivity for 4D segmentation.

In Figure 9, we note that the effect of temporal regularization is more prominent in some patients than in others; this is likely due to the amount of noise and/or the SNR in the choroid region for each particular image. If the temporal measurements obtained with 3D segmentation are reasonably consistent, there is no reason for the 4D measurements to be any different. This also highlights the fact that our 4D method is not biased toward a smaller temporal change. Because the temporal regularization is achieved with “hard” constraints on maximum allowed displacement rather than “soft” constraints that penalize larger displacements (such as the approach proposed in Ref. 9), the temporal sensitivity of the method is preserved.

Clearly, to maximize segmentation robustness of the joint longitudinal segmentation approach, availability of more than two time points would be beneficial. Nevertheless, it is noteworthy that the 4D segmentation enjoys substantial performance improvement compared with the cross-sectional 3D segmentation even with only two time points available, as demonstrated in the reproducibility experiments.

By definition, the presented algorithm cannot successfully segment sequences of images that contain larger thickness changes than what the hard constraints used in the graph construction to limit temporal change allow. We found that the value of 72.8 μm was a generous allowance to capture the choroidal changes observed in AMD. However, this parameter may need to be adjusted if the algorithm is applied to studies with dramatic choroidal changes. Additionally, for studies that involve either very long or uneven intervals between time points, it may be beneficial to make this parameter a function of the time elapsed rather than a constant number.

A possible limitation of the algorithm is that it would not be able to recover from a catastrophic failure in the initialization step. However, the BM segmentation algorithm has been previously thoroughly validated and such a major failure is therefore unlikely.

The longitudinal choroid segmentation presented here is the first application of our 4D graph-based segmentation method. Because the choroid region typically has an especially low SNR, the improvement achieved by the proposed 4D segmentation method is very pronounced. Nevertheless, we expect our approach to maintain its superior performance when segmenting intraretinal layers, which is an extension that remains as future work. Additionally, the relatively flat geometry of the choroid makes it straightforward to carry out synthetic experiments that are crucial for validating the reproducibility and sensitivity of this approach.
